# Supplementary material for: Hand hygiene after the COVID-19 pandemic: Is it still at a high level?
Source: PLoS One. 2025 Sep 19;20(9):e0332634. doi: 10.1371/journal.pone.0332634 (PMC12448956; doi:10.1371/journal.pone.0332634)
Supplement: S1 Table — (PDF) [file pone.0332634.s001.pdf]

S1 Table. Observation values and compliance of two phases

Phase	Month	No. of observations	No. of observations where compliance with HH was recorded	HH compliance, % (95% CI)	Mean HH compliance, % (95% CI)
Phase 1	Oct.2021	48	46	95.83 (85.75 to 99.49)	90.27 (85.34 to 89.54)
	Nov.2021	135	123	91.11 (84.99 to 95.32)	
	Dec.2021	70	61	87.14 (76.99 to 93.95)	
	Jan.2022	80	66	82.50 (72.38 to 90.09)	
	Feb.2022	98	88	89.80 (82.03 to 95.00)	
	Mar.2022	36	33	91.67 (77.53 to 98.25)	
	Apr.2022	62	53	85.48 (74.22 to 93.14)	
	May2022	63	54	85.71 (74.61 to 93.25)	

	Jun.2022	51	45	88.24 (78.59 to 96.74)	
	Jul.2022	73	70	95.89 (88.46 to 99.14)	
	Aug.2022	48	43	89.58 (77.34 to 96.53)	
	Sep.2022	90	87	96.67 (90.57 to 99.31)	
	Oct.2022	65	59	90.77 (80.98 to 96.54)	
	Nov.2022	22	19	86.36 (65.09 to 97.09)	
	Dec.2022	11	11	100.00 (71.51 to 100.00)	
	Jan.2023	14	14	100.00 (76.84 to 100.00)	
	Feb.2023	36	32	88.89 (73.94 to 96.89)	82.56 (80.35 to 84.61)
	Mar.2023	43	32	74.42 (58.83 to 86.48)	
	Apr.2023	17	13	76.47 (50.10 to 93.19)	
	May2023	74	59	79.73 (68.78 to 88.19)	
	Jun.2023	148	127	85.81 (79.13 to 91.00)	

Phase 2	Jul.2023	110	84	76.36 (67.32 to 83.94)	
	Aug.2023	105	89	84.76 (76.44 to 91.03)	
	Sep.2023	71	61	85.92 (75.62 to 93.03)	
	Oct.2023	42	35	83.33 (68.64 to 93.03)	
	Nov.2023	46	40	86.96 (73.74 to 95.06)	
	Dec.2023	85	66	77.65 (67.31 to 85.97)	
	Jan.2024	62	52	83.87 (72.33 to 91.98)	
	Feb.2024	40	33	82.50 (67.22 to 92.66)	
	Mar.2024	100	84	84.00 (75.32 to 90.57)	
	Apr.2024	80	65	81.25 (70.97 to 89.11)	
	May2024	79	63	79.75 (69.20 to 87.96)	
	Jun.2024	51	44	86.27 (73.74 to 94.30)	
	Jul.2024	78	67	85.90 (76.17 to 92.74)	

total		1918	2233	85.89 (84.38 to 87.31)	
