# Supplementary figures and images for: Hand hygiene after the COVID-19 pandemic: Is it still at a high level?
Source: PLoS One. 2025 Sep 19;20(9):e0332634. doi: 10.1371/journal.pone.0332634 (PMC12448956; doi:10.1371/journal.pone.0332634)

**S1 Figure. Change in overall HH compliance between Phase 1 and Phase 2.**

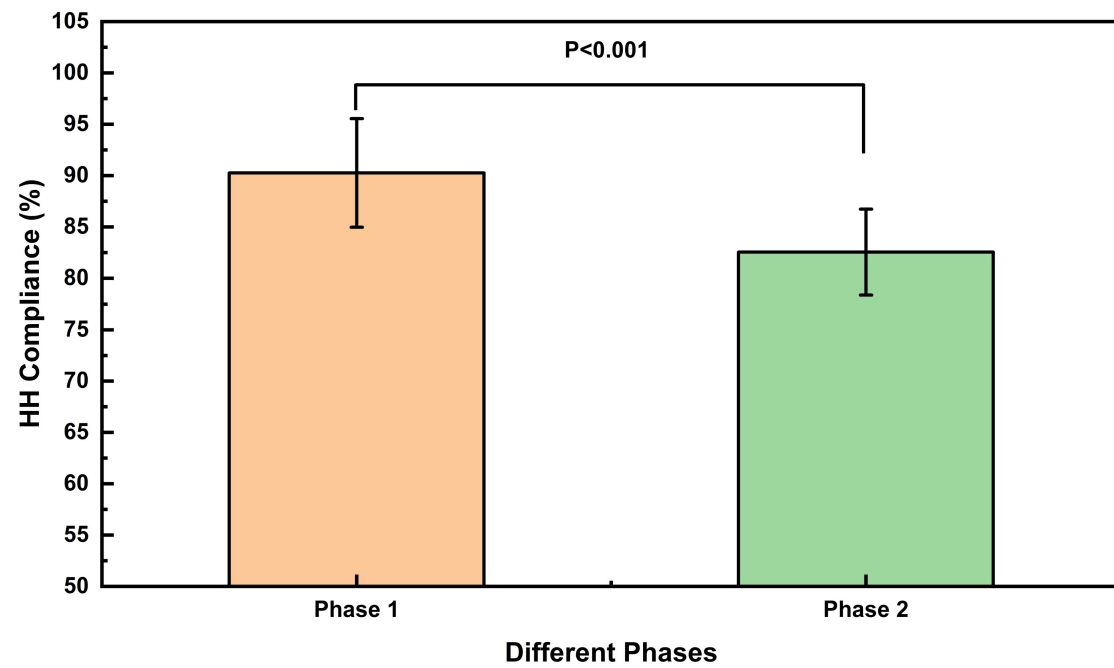

Supplement: S1 Fig — (PDF) [file pone.0332634.s002.pdf]
