# Supplementary material for: Hand hygiene after the COVID-19 pandemic: Is it still at a high level?
Source: PLoS One. 2025 Sep 19;20(9):e0332634. doi: 10.1371/journal.pone.0332634 (PMC12448956; doi:10.1371/journal.pone.0332634)
Supplement: S2 Table — (PDF) [file pone.0332634.s003.pdf]

S2 Table. Observation values and compliance for different healthcare worker groups

Phase	Doctor			Nurse			Paramedical staff			Cleaning staff		
	HHA	HHO	Comp% (95% CI)	HHA	HHO	Comp% (95% CI)	HHA	HHO	Comp% (95% CI)	HHA	HHO	Comp% (95% CI)
Phase 1	131	156	83.97 (77.25 to 89.35)	690	746	92.49 (90.36 to 94.28)	37	45	82.22 (67.95 to 92.00)	14	19	73.68 (48.80 to 90.85)
Phase 2	167	248	67.34 (61.12 to 73.14)	815	932	87.45 (85.15 to 89.51)	43	63	68.25 (55.31 to 79.42)	21	24	87.50 (67.64 to 97.34)
total	298	404	73.76 (69.18 to 77.99)	1505	1678	89.69 (88.14 to 91.10)	80	108	74.07 (64.75 to 82.03)	35	43	81.40 (66.60 to 91.61)

HHA= hand hygiene action, HHO= hand hygiene opportunity, Comp%= hand hygiene compliance (%).
