# Supplementary material for: Hand hygiene after the COVID-19 pandemic: Is it still at a high level?
Source: PLoS One. 2025 Sep 19;20(9):e0332634. doi: 10.1371/journal.pone.0332634 (PMC12448956; doi:10.1371/journal.pone.0332634)
Supplement: S3 Table — (PDF) [file pone.0332634.s004.pdf]

S3 Table. Observation values and compliance concerning "Your five moments for hand hygiene" (WHO)

Phase	M1			M2			M3-1			M3			M4			M5		
	HH A	HH O	Comp% (95% CI)	HH A	HH O	Comp% (95% CI)	HH A	HH O	Comp% (95% CI)	HH A	HH O	Comp% (95% CI)	HH A	HH O	Comp% (95% CI)	HH A	HH O	Comp% (95% CI)
Phase 1	25	27	92.59 (75.71 to 99.09)	57	62	91.94 (82.17 to 97.33)	5	5	100.00 (47.82 to 100.00)	27	35	77.14 (59.86 to 89.58)	488	536	91.04 (88.30 to 93.32)	275	306	89.87 (85.93 to 93.01)
Phase 2	49	65	75.38 (63.13 to 85.23)	69	86	80.23 (70.25 to 88.04)	9	11	81.82 (48.22 to 97.72)	36	60	60.00 (46.54 to 72.44)	523	646	80.96 (77.72 to 83.92)	369	410	90.00 (86.68 to 92.73)
total	74	92	80.43 (70.85 to 87.97)	126	148	85.14 (78.36 to 90.44)	14	16	87.50 (61.65 to 98.45)	63	95	66.32 (55.89 to 75.69)	1011	1182	85.53 (83.40 to 87.49)	644	716	89.94 (87.50 to 92.05)

HHA= hand hygiene action, HHO= hand hygiene opportunity, Comp%= hand hygiene compliance (%). M1=Prior to patient contact; M2= Prior to a clean or aseptic procedure; M3-1=After contact with body fluid, excluding cases where gloves were worn; M3=After contact with body fluid; M4=After patient contact; M5=After contact with the patient environment.
