# Supplementary material for: Hand hygiene after the COVID-19 pandemic: Is it still at a high level?
Source: PLoS One. 2025 Sep 19;20(9):e0332634. doi: 10.1371/journal.pone.0332634 (PMC12448956; doi:10.1371/journal.pone.0332634)
Supplement: S2 Fig — (PDF) [file pone.0332634.s005.pdf]

S2 Figure. Changes in HH compliance for different moments after the instances of glove use being excluded during M3 (referred to as M3-1).

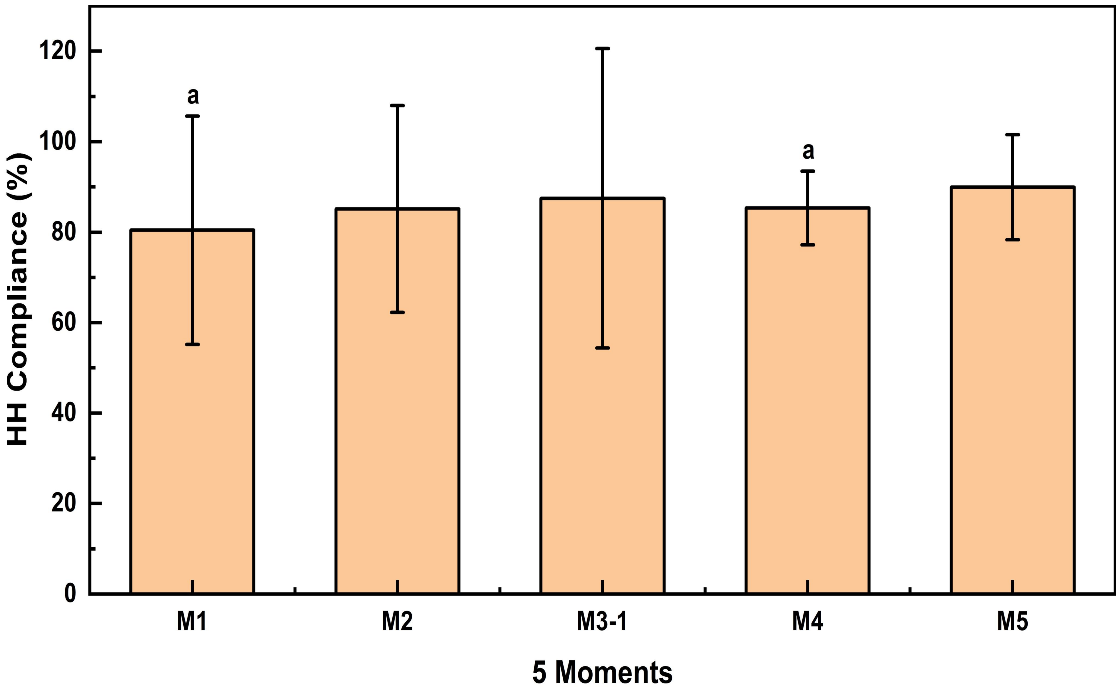

M1 : Before patient contact; M2 : Before clean/aseptic procedure; M3-1 : After body fluid exposure (excluding instances of glove use); M4 : After patient contact; M5 : After touching patient surroundings. a indicates a significant difference compared to M5 ($P < 0.05$)
