# Supplementary material for: Hand hygiene after the COVID-19 pandemic: Is it still at a high level?
Source: PLoS One. 2025 Sep 19;20(9):e0332634. doi: 10.1371/journal.pone.0332634 (PMC12448956; doi:10.1371/journal.pone.0332634)
Supplement: S4 Table — (PDF) [file pone.0332634.s006.pdf]

S4 Table. Observation values and compliance of different departments

Phase	Medical wards			Surgical wards			ICU			Operating room			Outpatient clinics			Other Units		
	HH A	HH O	Comp% (95% CI)	HH A	HH O	Comp% (95% CI)	HH A	HH O	Comp% (95% CI)	HH A	HH O	Comp% (95% CI)	HH A	HH O	Comp% (95% CI)	HH A	HH O	Comp% (95% CI)
Phase 1	242	279	86.74 (82.19 to 90.49)	198	224	88.39 (83.46 to 92.28)	126	133	94.74 (89.46 to 97.86)	35	37	94.59 (81.81 to 99.34)	168	180	93.33 (88.64 to 96.51)	103	113	91.15 (84.33 to 95.67)
Phase 2	250	303	82.51 (77.75 to 86.61)	239	301	79.40 (74.39 to 83.83)	182	199	91.46 (86.67 to 94.94)	34	38	89.47 (75.20 to 97.06)	217	269	80.67 (75.44 to 85.21)	124	157	78.98 (71.77 to 85.07)
total	492	582	84.54 (81.34 to 87.38)	437	525	83.24 (79.76 to 86.33)	308	332	92.77 (89.43 to 95.31)	69	75	92.00 (83.40 to 97.01)	385	449	85.75 (82.17 to 88.85)	227	270	84.07 (79.15 to 88.23)

HHA= hand hygiene action, HHO= hand hygiene opportunity, Comp%= hand hygiene compliance (%).
