# Supplementary material for: Hand hygiene after the COVID-19 pandemic: Is it still at a high level?
Source: PLoS One. 2025 Sep 19;20(9):e0332634. doi: 10.1371/journal.pone.0332634 (PMC12448956; doi:10.1371/journal.pone.0332634)
Supplement: S3 Fig — (PDF) [file pone.0332634.s007.pdf]

S3 Figure. Comparison of HH compliance (a) between Phase 1and Phase 2 among different seasons and Changes in HH compliance (b) for different seasons.

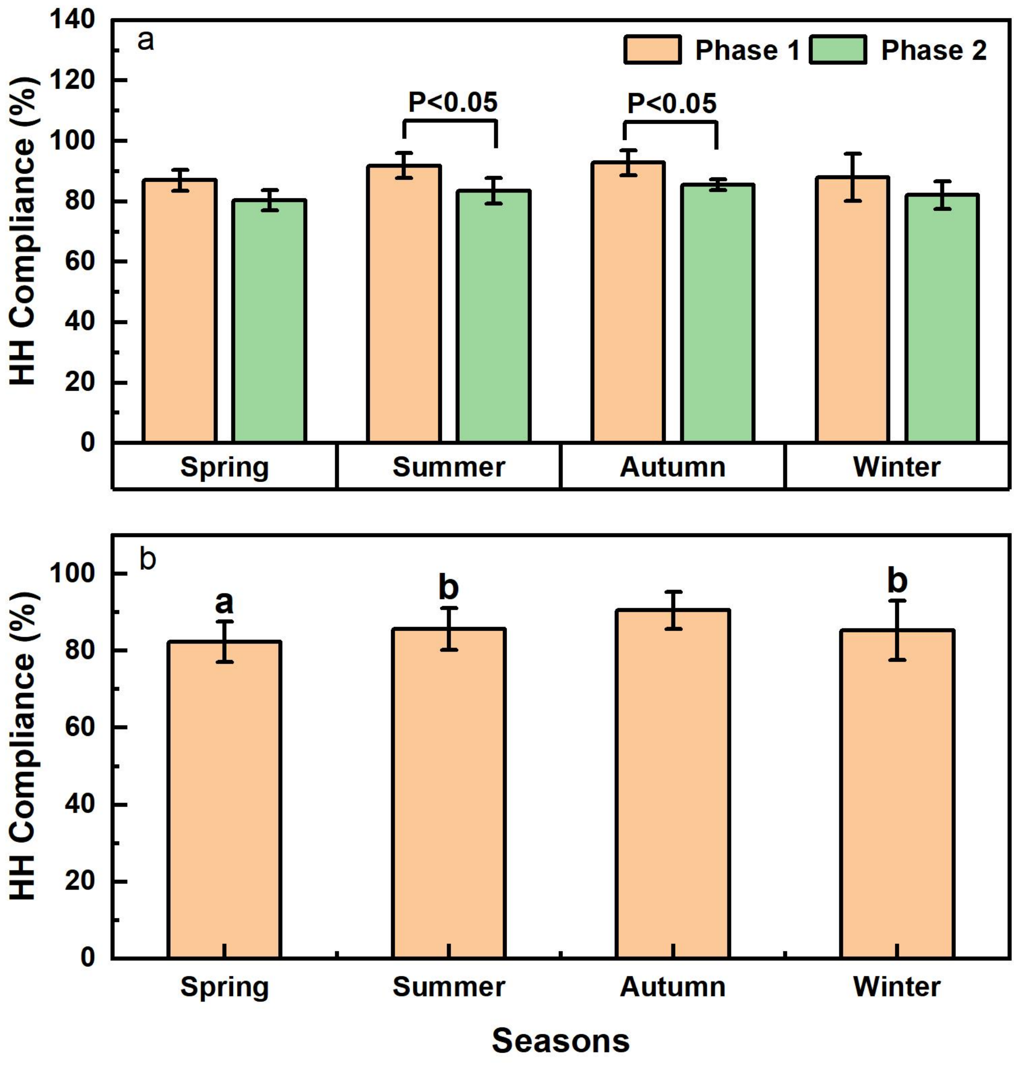

a indicates a significant difference compared to Autumn (*P* < 0.001). b indicates a significant difference compared to Autumn (*P* < 0.05).
