# Supplementary material for: Hand hygiene after the COVID-19 pandemic: Is it still at a high level?
Source: PLoS One. 2025 Sep 19;20(9):e0332634. doi: 10.1371/journal.pone.0332634 (PMC12448956; doi:10.1371/journal.pone.0332634)
Supplement: S5 Table — (PDF) [file pone.0332634.s008.pdf]

S5 Table. Observation values and compliance of different seasons

Phase	Spring			Summer			Autumn			Winter		
	HHA	HHO	Comp% (95% CI)	HHA	HHO	Comp% (95% CI)	HHA	HHO	Comp% (95% CI)	HHA	HHO	Comp% (95% CI)
Phase 1	140	161	86.96 (80.76 to 91.74)	158	172	91.86 (86.72 to 95.48)	334	360	92.78 (89.60 to 95.23)	240	273	87.91 (83.44 to 91.53)
Phase 2	316	393	80.41 (76.13 to 84.22)	411	492	83.54 (79.96 to 86.71)	136	159	85.53 (79.09 to 90.60)	183	223	82.06 (76.39 to 86.87)
total	456	554	82.31 (78.87 to 85.40)	569	664	85.69 (82.80 to 88.27)	470	519	90.56 (87.71 to 92.93)	423	496	85.28 (81.85 to 88.28)

HHA= hand hygiene action, HHO= hand hygiene opportunity, Comp%= hand hygiene compliance (%).
