# Supplementary material for: Hand hygiene after the COVID-19 pandemic: Is it still at a high level?
Source: PLoS One. 2025 Sep 19;20(9):e0332634. doi: 10.1371/journal.pone.0332634 (PMC12448956; doi:10.1371/journal.pone.0332634)
Supplement: S6 Table — (PDF) [file pone.0332634.s009.pdf]

S6 Table. Observation values and incidence of Hand-rubbing time>15 s

Phase	ABHR			Soup and water			Total		
	HRD>15 s	OHHO	Incide% (95% CI)	HRD>15 s	OHHO	Incide% (95% CI)	HRD>15 s	OHHO	Incide% (95% CI)
Phase 1	554	603	91.87 (89.33 to 93.87)	259	269	96.28 (93.06 to 98.10)	813	872	93.23 (91.31 to 94.77)
Phase 2	644	716	89.94 (87.45 to 92.00)	307	330	93.03 (89.58 to 95.43)	951	1046	90.92 (88.97 to 92.56)
total	1198	1319	90.83 (89.11 to 92.30)	566	599	94.49 (92.27 to 96.12)	1764	1918	91.97 (90.64 to 93.13)

ABHR: alcohol-based hand rubs, HRD: Hand-rubbing duration, OHHO: overall hand hygiene observation. Incide%: Incidence of hand-rubbing time >15s
