# Supplementary material for: Hand hygiene after the COVID-19 pandemic: Is it still at a high level?
Source: PLoS One. 2025 Sep 19;20(9):e0332634. doi: 10.1371/journal.pone.0332634 (PMC12448956; doi:10.1371/journal.pone.0332634)

S4 Figure. Incidence of hand-rubbing duration >15 s (a) for different phases and (b) different methods (ABHR and soup &water)

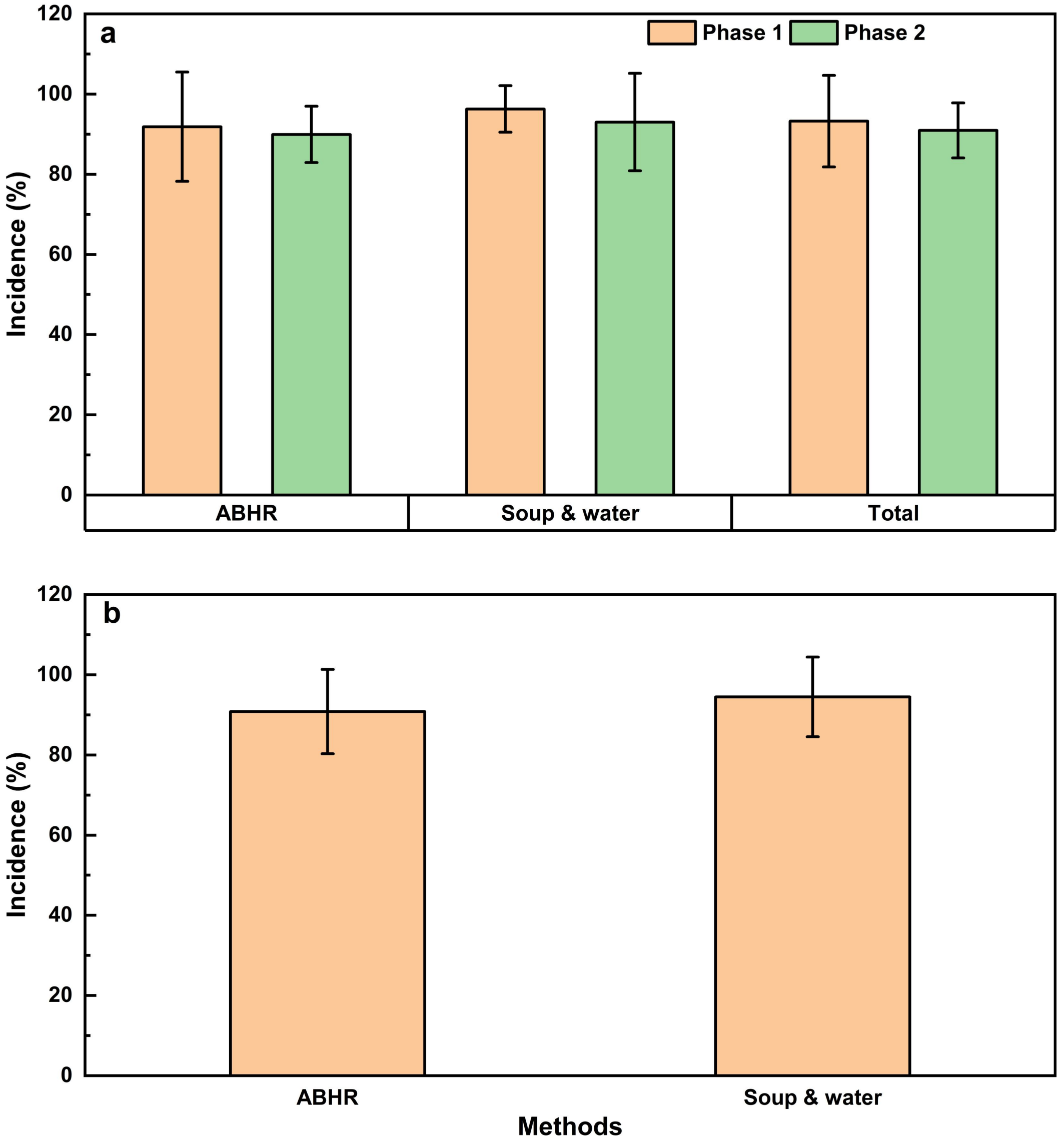

Supplement: S4 Fig — (PDF) [file pone.0332634.s010.pdf]
