# Supplementary material for: Hand hygiene after the COVID-19 pandemic: Is it still at a high level?
Source: PLoS One. 2025 Sep 19;20(9):e0332634. doi: 10.1371/journal.pone.0332634 (PMC12448956; doi:10.1371/journal.pone.0332634)
Supplement: S7 Table — (PDF) [file pone.0332634.s011.pdf]

S7 Table. Distribution of HAIs in two phases

HAI type	Phase 1				Phase 2				OR	95% CI	*P*-value
	No. of inpatients	No. of HAIs	Incidence (‰)	95%CI	No. of inpatients	No. of HAIs	Incidence (‰)	95%CI			
LRTI	179651	1422	7.92	7.51 to 8.34	262421	2348	8.95	8.59 to 9.32	1.13	1.06 to 1.21	<0.001
UTI	179651	545	3.03	2.78 to 3.30	262421	726	2.77	2.57 to 2.98	0.91	0.82 to 1.02	0.1033
APTI	179651	321	1.79	1.60 to 1.99	262421	421	1.60	1.45 to 1.76	0.90	0.78 to 1.04	0.1454
URI	179651	225	1.25	1.09 to 1.43	262421	628	2.39	2.21 to 2.59	1.91	1.64 to 2.23	<0.001

SST	179651	88	0.49	0.39 to 0.60	262421	114	0.43	0.36 to 0.52	0.89	0.67 to 1.17	0.3971
GI	179651	85	0.47	0.38 to 0.59	262421	153	0.58	0.49 to 0.68	1.23	0.95 to 1.61	0.1218
SSI	179651	92	0.51	0.41 to 0.63	262421	160	0.61	0.52 to 0.71	1.19	0.92 to 1.54	0.1818
BSI	179651	81	0.45	0.36 to 0.56	262421	114	0.43	0.36 to 0.52	0.96	0.72 to 1.28	0.7981
EENT	179651	31	0.17	0.12 to 0.24	262421	97	0.37	0.30 to 0.45	2.14	1.43 to 3.21	<0.001
CAUTI	179651	25	0.14	0.09 to 0.21	262421	32	0.12	0.08 to 0.17	0.88	0.52 to 1.48	0.6205
VAP	179651	23	0.13	0.08 to	262421	30	0.11	0.08 to	0.89	0.52 to	

				0.19				0.16		1.54	0.6827
CLABSI	179651	17	0.09	0.06 to 0.15	262421	17	0.06	0.04 to 0.10	0.68	0.35 to 1.34	0.2664
Others	179651	61	0.34	0.26 to 0.44	262421	70	0.27	0.21 to 0.34	0.79	0.56 to 1.11	0.1672
All HCAIs	179651	3016	16.79	16.20 to 17.39	262421	4910	18.71	18.20 to 19.24	1.12	1.07 to 1.17	<0.001

OR: odds ratio, the HAI in phase 1 as a reference; CI: confidence interval.

HAI: healthcare associated infections, LRTI=lower respiratory tract infection, SSI= surgical site infection, SST=skin and soft tissue infection, UTI= urinary tract infection, BSI=bloodstream infection, CLABSI=central line-associated bloodstream infection, URI=upper respiratory infection, GI =gastrointestinal infection, EENT= eye, ear, nose, throat or mouth infection, CAUTI=catheter-associated urinary tract infections, VAP=ventilator-associated pneumonia, APTI= Abdominal and pelvic tissue infection，Others=cardiovascular infections, bone infections, central nervous system infections, reproductive tract infection.
